# Scaling the Andean Shilajit: A Novel Neuroprotective Agent for Alzheimer’s Disease

**DOI:** 10.3390/ph16070960

**Published:** 2023-07-04

**Authors:** Víctor Andrade, Maylin Wong-Guerra, Nicole Cortés, Gabriela Pastor, Andrea González, Camila Calfío, Leonardo Guzmán-Martínez, Leonardo P. Navarrete, Nicolas Ramos-Escobar, Inelia Morales, Rocío Santander, Juan Andrades-Lagos, Mitchell Bacho, Leonel E. Rojo, Ricardo Benjamín Maccioni

**Affiliations:** 1 Laboratory of Neuroscience and Functional Medicine, International Center for Biomedicine, Faculty of Sciences, University of Chile, Santiago 7800003, ChileInelia.morales@gmail.com (I.M.); 2 Division of Neurogenetics and Molecular Psychiatry, Department of Psychiatry and Psychotherapy, Medical Faculty, University of Cologne, 50923 Köln, Germany; 3 Department of Neurodegenerative Diseases and Geriatric Psychiatry, University Hospital Bonn, 53127 Bonn, Germany; 4 Laboratory of Toxicology and Metabolism, Faculty of Chemistry and Biology, University of Santiago of Chile, Santiago 9170022, Chile; 5 Biochemistry School, Faculty of Health Sciences, Andres Bello University, Santiago 8370035, Chile; 6 Laboratory of Kinetics and Photochemistry, Faculty of Chemistry and Biology, University of Santiago of Chile, Santiago 9170022, Chile; rocio.santanderm@usach.cl; 7 Facultad de Medicina y Ciencia, Universidad San Sebastián, Santiago 7510157, Chile; juan.andrades@uss.cl; 8 Drug Development Laboratory, Faculty of Chemical and Pharmaceutical Sciences, University of Chile, Santiago 8380492, Chile; 9 Organic and Organometallic Synthesis Laboratory, Faculty of Chemistry, Andrés Bello University, Santiago 8370186, Chile; mbachol@ciq.uchile.cl; 10 Laboratory of Natural Resources, Faculty of Sciences, University of Chile, Santiago 7750000, Chile

**Keywords:** Alzheimer’s disease (AD), Andean Shilajit (AnSh), bioactive fractions, molecular networks, neuroprotector, prevalent neurological disorders

## Abstract

Alzheimer’s disease (AD) is a multifactorial neurodegenerative disorder without a cure, despite the enormous number of investigations and therapeutic approaches. AD is a consequence of microglial responses to “damage signals”, such as aggregated tau oligomers, which trigger a neuro-inflammatory reaction, promoting the misfolding of cytoskeleton structure. Since AD is the most prevalent cause of dementia in the elderly (>60 years old), new treatments are essential to improve the well-being of affected subjects. The pharmaceutical industry has not developed new drugs with efficacy for controlling AD. In this context, major attention has been given to nutraceuticals and novel bioactive compounds, such as molecules from the Andean Shilajit (AnSh), obtained from the Andes of Chile. Primary cultures of rat hippocampal neurons and mouse neuroblastoma cells were evaluated to examine the functional and neuroprotective role of different AnSh fractions. Our findings show that AnSh fractions increase the number and length of neuronal processes at a differential dose. All fractions were viable in neurons. The AnSh fractions inhibit tau self-aggregation after 10 days of treatment. Finally, we identified two candidate molecules in M3 fractions assayed by UPLC/MS. Our research points to a novel AnSh-derived fraction that is helpful in AD. Intensive work toward elucidation of the molecular mechanisms is being carried out. AnSh is an alternative for AD treatment or as a coadjuvant for an effective treatment.

## 1. Introduction

Alzheimer’s disease (AD) is the most prevalent type of dementia in the population over 60 years [1,2], according to the World Health Organization (WHO) [3], and gradually affects learning and memory. Furthermore, our laboratory recently associated this pathology with the presence of behavioral symptoms occurring before full-blown cognitive impairment [4]. This expansive and epidemic brain disorder has become an issue of major concern to the medical community and public health authorities, and thus significant efforts are underway, focused on its prevention and treatment.

In the biological context, two main etiological effectors have been identified for AD: (i) Neurofibrillary tangles (NFT), derived from the progressive aggregation of the hyperphosphorylated protein tau inside the neurons, assembled in oligomeric structures referred to as paired helical filaments (PHF) [5,6,7,8]; and (ii) senile plaques (SP) formed by deposits of the amyloid-β (Aβ) peptide (39 to 42 aminoacidic residues). Amyloid aggregates come from the proteolytic excision of the amyloid precursor protein (APP) [1,9]. Both aggregates promote the loss of synaptic processes and neuronal death [1,9]. We proposed that the onset of AD is a consequence of the response of microglial cells to "damage signals" or tau oligomers, which trigger a neuroinflammatory response, promoting the unfolding of the cytoskeletal structure [7,10,11]. This perspective is of great importance, as it opens a feasible avenue for the early prevention of neuro-inflammation in AD pathology. The pharmaceutical industry has failed to develop new efficacious drugs for controlling AD.

Since AD is a multifactorial disease, new preventive strategies are being implemented to prevent AD based on changes in diet and nutritional supplements, functional foods, and natural compounds [12,13,14,15]. The use of natural bioactive molecules against AD is a topic that has been researched by several groups [16], as well as ours, suggesting that natural multicomponent formulas have shown efficacy against AD [17,18]. Since AD is currently considered a multifactorial disease, this approach is promising. The antioxidant properties of several beneficial nutraceuticals, such as curcumin, honey, quercetin, and berries, among others, are also well documented and could modify AD pathology [19].

Most nutraceuticals have polyphenols as their main components, showing metal-chelating and anti-inflammatory properties [20,21]. Thus, it is of interest to further research novel multitarget therapies from natural sources.

In this context, considerable attention has been given to clinically validated nutraceuticals and novel bioactive compounds, such as the Andean Shilajit (AnSh), obtained from the northern Chilean Andes. This is a fossilized product resulting from the millenary degradation of plant material by the action of several types of microbial agents. AnSh is rich in humins, including fulvic acids (FA), humic acids (HA), and some inorganic molecules such as selenium, magnesium, and other minerals [12,22,23]. BrainUp-10^®^ is a new formulation based on AnSh and supplemented with B vitamin complex [12,22,23]. Preliminary studies suggest that BrainUp-10^®^ and AnSh can achieve effectiveness at controlling the disease [12,22]. Intensive work is being carried out to understand the molecular mechanisms of action of the AnSh, which we further describe in this work. Recent clinical studies have demonstrated the efficacy of the AnSh against AD [24]. 

In this study, we aim to describe the mode of action of AnSh, focusing on its neuritogenic and anti-tau aggregation properties. The adult brain can change its structure and function during development and learning, or as a result of environmental changes or pathological conditions, for which neuritogenesis is necessary [25]. Recent studies have proposed that the development of the dendritic tree allows newborn neurons to communicate with each other through circuit binding [26], while in adulthood, neuritogenesis, the formation of neuronal polarity, and the maturation of axons and dendrites are highly involved in the processes of synaptic plasticity, memory and learning [27,28,29]. These processes can be affected by neurodegenerative disorders [29,30]. Therefore, interest in novel drugs that improve neuritogenesis in regions affected by neurodegenerative diseases like AD has been growing in an effort to support deficiency related to cell death, which plays a central role in a wide variety of CNS disorders. To study the main components of the AnSh responsible for the observed effect, fractions of the AnSh will also be evaluated.

The present study investigates the effect of AnSh and its fractions on tau protein aggregation and neuritogenesis. The context of our study focuses on the action mode of AnSh and the identification, for the first time, of novel fractions and bioactive molecules with potential neuroprotective and restorative mechanisms which are stronger than the effects observed in our previous reports, providing new evidence on the chemistry of AnSh. All analyses were performed using cultured neuronal cells and in vitro cell-free assays. 

## 2. Results

### 2.1. Chemical Investigation of the Andean Shilajit

**Figure 1:**
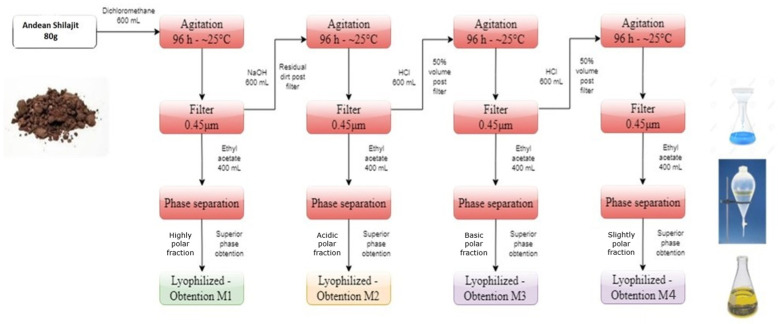
**Main fractions extracted from the Andean Shilajit.** The diagram presents the polarity-based fractionation process for the M1, M2, M3, and M4 fractions.

The AnSh was subjected to a chemical separation to obtain several fractions and subfractions, according to their properties (Figure 1). Briefly, four main fractions derived from the treatment of the AnSh were extracted according to their polarity and precipitation capabilities. Later, three subfractions were also extracted from the fraction M3 due to their strong effects on neurons. Starting from 80 g of Andean Shilajit, based on previous research [31,32], M1, which contains mainly organic and polar molecules, M2, composed mainly of molecules with acid properties, M3, composed mostly of basic molecules, and, M4, which is slightly polar, were extracted. The M3 subfractions were separated by polarity, using a chromatography column after precipitation (3 days).

### 2.2. Andean Shilajit Fractions Are Not Toxic in N2a Cells

**Figure 2:**
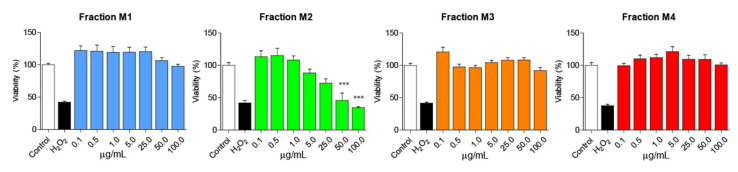
**N2a cells are viable after treatment with the different fractions of Andean Shilajit.** Concentrations from 0.1 μg/mL to 100 μg/mL of each fraction of the AnSh (*n* = 4–5) were tested in N2a cells. Only high concentrations (50 and 100 μg/mL) of the M2 fraction were toxic to cells. ANOVA test, factor followed by Tukey’s post hoc test. *** = *p*-value < 0.001.

To determine the cytotoxic effect of AnSh, MTT reduction assays were performed on N2a cells, using the four different fractions (M1, M2, M3, and M4). We found that different concentrations of the fractions did not induce significant impairment in N2a cell viability (Figure 2), even at 100 μg/mL. The M2 fraction was toxic to N2a cells only in the high concentration range (50 and 100 μg/mL), with cell viability reduced by up to 50%, *p* < 0.001 (Figure 2), compared to the control group after treatment for 24 h, with significant differences with respect to the H_2_O_2_-treated group. Concentrations of 0.5; 1.0; 5.0 and 25 μg/mL of each of the fractions were used for the subsequent studies, considering their relatively low toxicity.

### 2.3. Andean Shilajit Fractions Increase Neuritogenesis and the Number of Neuronal Processes in N2a Cells

**Figure 3:**
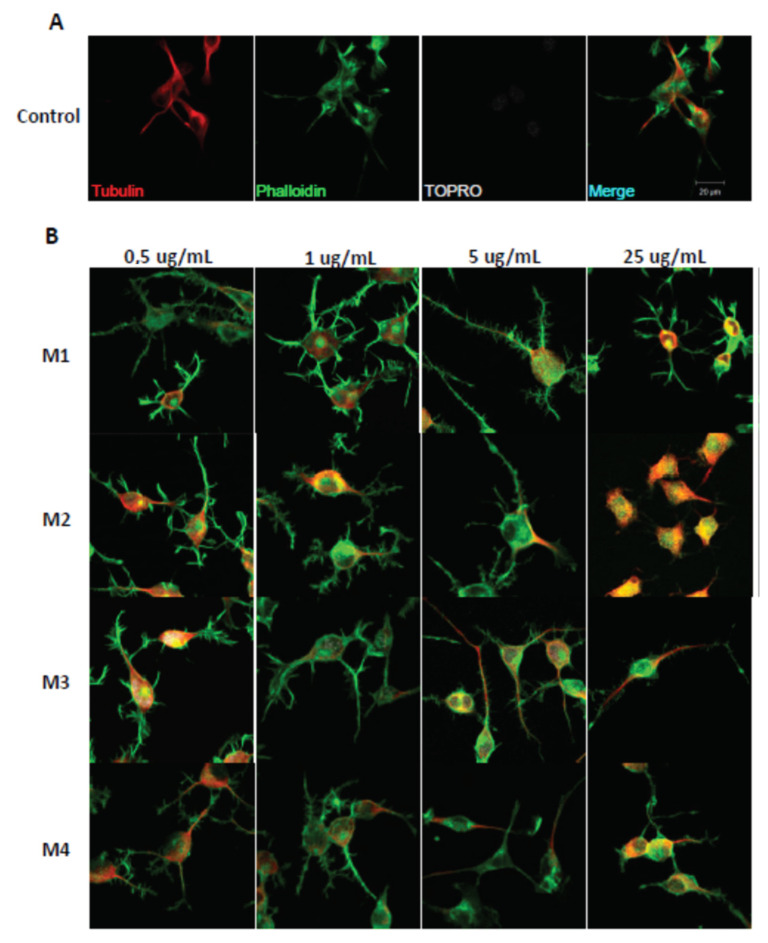
**Immunohistochemical staining of N2a cells treated with M1, M2, M3, and M4 fractions for 24 h.** Immunofluorescence assays representing the control condition (**A**). Protein markers used in the experiment are shown as follows: α-Tubulin: microtubule-associated protein marker; Phalloidin: β-actin protein marker; Topro: cell core, and merge of all the channels involved. (**B**) Representative images of N2a cells treated with 0.5 μg/mL, 1.0 μg/mL, 5.0 μg/mL, and 25.0 μg/mL of AnSh fractions.

**Figure 4:**
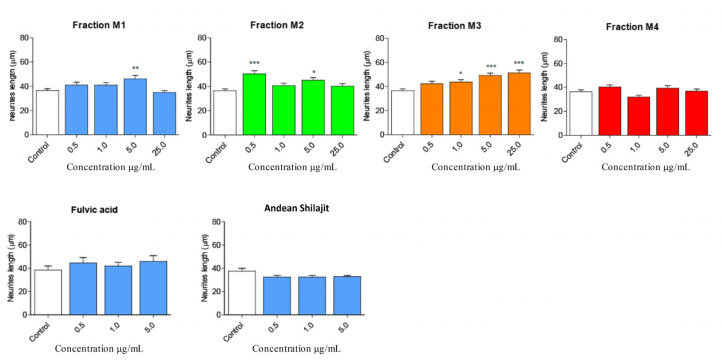
**Effect of AnSh fractions on the length of neurites in N2a cells.** A wide range of concentrations of AnSh fractions morphologically modifies N2a cells. Concentrations from 0.5 μg/mL to 25 μg/mL of each AnSh fraction in N2a cells (*n* = 4–5), and up to 5 μg/mL of fulvic acid and total Andean Shilajit (*n* = 3) were tested. The values represent the standard error. ANOVA test, followed by Tukey’s post hoc test. * = *p* value < 0.05, ** = *p* value < 0.01, *** = *p* value < 0.001.

**Figure 5:**
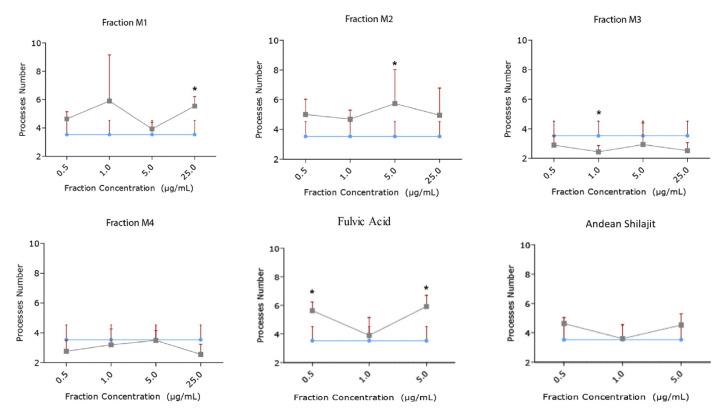
**Effect of AnSh fractions on the number of neuritogenic processes (number of neurites) in N2a cells**. Concentrations from 0.5 μg/mL to 25 μg/mL of each AnSh fraction (*n* = 4–5) in N2a cells, and up to 5 μg/mL of fulvic acid and total Andean Shilajit (*n* = 3) were tested. The values represent the standard error. ANOVA test, followed by Tukey’s post hoc test * = *p* value < 0.05.

The immunofluorescence assays showed the effect of the different fractions on the morphology of the N2a cells, specifically the length of the neurites and the number of processes, as part of the neuritogenic process. The different treatments, in general, promoted morphological changes in N2a cells, resulting in an increase in their length and number of processes (Figure 3). The M1 fraction increased the length of the neurites at a concentration of 5 μg/mL (Figure 4), likewise, M1 increased the number of processes by 37% at a concentration of 25 μg/mL (Figure 5). The M2 fraction increased the length of the neurites at concentrations of 0.5 (38.54%) and 5 μg/mL (24.36%) (Figure 4), while 5.0 μg/mL significantly increased the number of processes, by 62.83% (Figure 5) (Table 1). 

Moreover, the M3 fraction significantly increased the length of the neurites in a concentration-dependent manner, from 1 μg/mL to 25 μg/mL, with reference to the control (Figure 4). At a concentration of 1.0 μg/mL, M3 increased the process length by 20.42%, and at 5.0 μg/mL, by 35.23%. Meanwhile, M3 at 25.0 μg/mL increased the process length by 41% (Figure 5). According to our results, M3 did not increase the number of processes in N2a cells compared with the control condition (Figure 4). The M4 fraction did not modify the neurite length or the number of processes of N2a cells compared with the control group (Figure 4 and Figure 5).

In addition, the effects of fulvic acid and the AnSh were evaluated. None of the tested molecules had a significant impact on neurite growth (Figure 4); however, treatment with fulvic acid significantly increased the number of processes at concentrations of 0.5 μg/mL (59.91%) and 5.0 μg/mL (68.07%) (Figure 5) (Table 1).

**Figure 6:**
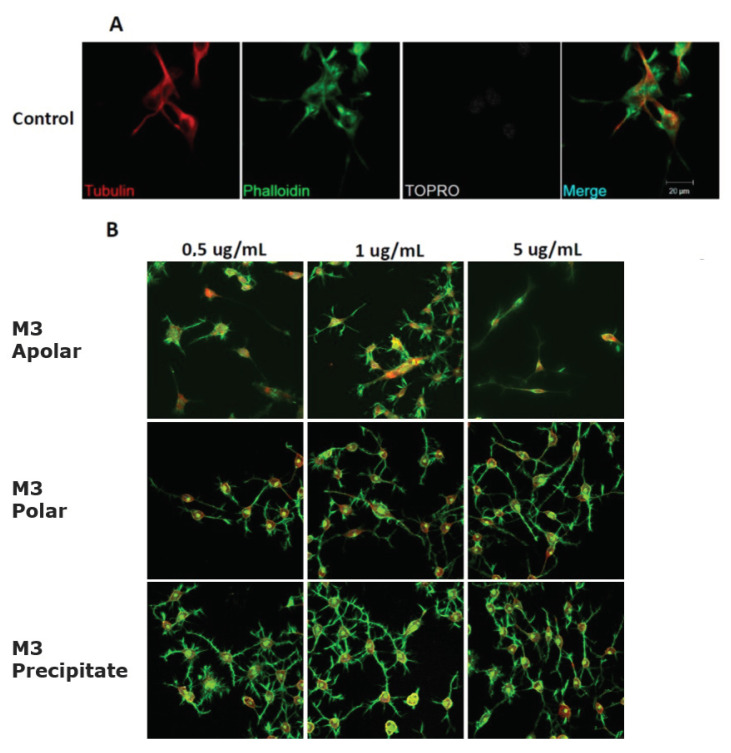
Representative morphology images of N2a cells after treatment with different concentrations of the M3 subfractions. Immunofluorescence assays represent different assay conditions. (**A**) Control condition. Protein markers used in the experiment are shown as follows: α-Tubulin: microtubule-associated protein marker; Phalloidin: β-actin protein marker; Topro: cell core, and merge of all the channels involved. (**B**) The representative images of N2a cells present their morphological changes after being treated with 0.5 μg/mL, 1.0 μg/mL, and 5.0 μg/mL of the M3 subfractions for 24 h.

**Figure 7:**
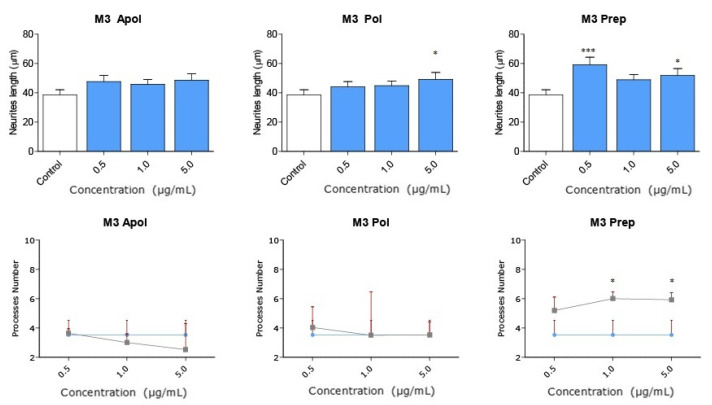
**Subfractions of M3 modulate N2a morphology at a wide range of concentrations.** Concentrations from 0.1 μg/mL to 5.0 μg/mL of each subfraction of M3 were tested in N2a cells (*n* = 3). All concentrations of Precipitate M3 subfraction elicited morphological changes (length and the number of neurites) in N2a cells after 24 h. Only 5.0 μg/mL of Polar M3 increased the length of neurites. Values represent the standard error. ANOVA test, factor followed by post hoc Tukey test. * = *p*-value < 0.05, *** = *p*-value < 0.001.

Considering the strong neuritogenic effect of the M3 fraction, we aimed to further study three M3 subfractions of the AnSh to identify the purified fractions responsible for this effect. For this purpose, the effects of apolar (Apolar M3), polar (Polar M3), and precipitate (Precipitate M3) subfractions were evaluated in N2a cells after treatment for 24 h (Figure 6 and Figure 7). The concentrations of 0.5 μg/mL and 5 μg/mL of the subfraction of Precipitate M3 resulted in a morphological modulation of the length of neurites, by 63.20% and 42.61%, respectively. Meanwhile, the concentrations of 1 μg /mL and 5 μg/mL of Precipitate M3 showed a significant increase in the number of neurites, 70.22% and 68.07%, respectively, after 24 h of treatment (Figure 6 and Figure 7) (Table 1). Only 5.0 μg/mL of Polar M3 increased the length of neurites, while having no effect on the number of neuronal processes. Apolar M3 had no significant effect on the morphological parameters evaluated in N2a cells (Figure 6 and Figure 7) (Table 1). Therefore, our results suggest that Precipitate M3 induces the strongest coordinated neuritogenic effect on the length and number of neurites of N2a cells.

### 2.4. The Precipitate M3 Subfraction and BrainUp-10 ^®^ Increase Neuritogenesis and the Number of Processes in Rat Hippocampal Neurons (RHN)

**Figure 8:**
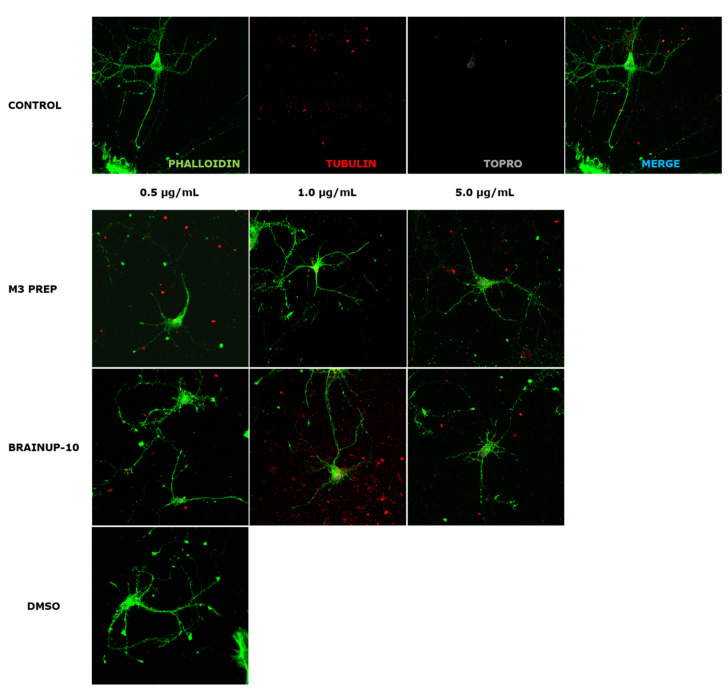
**Representative morphology images of RHN cells after treatment with different concentrations of the Precipitate M3 subfraction or BrainUp-10^®^.** Immunofluorescence assays represent different assay conditions. Control condition. Protein markers used in the experiment are shown as follows: Phalloidin: β-actin protein marker; α-Tubulin: microtubule-associated protein marker; Topro: cell core and merge of all the channels involved. The representative images of RHN present morphological changes after being treated with 0.5 μg/and mL; 1.0 μg/mL, 5.0 μg/mL of the Precipitate M3 subfraction or BrainUp-10^®^ for 24 h. DMSO is shown as the vehicle solution control.

**Figure 9:**
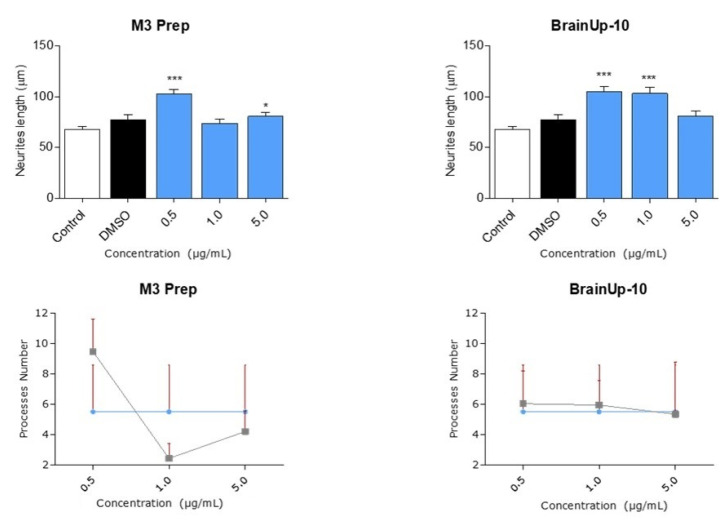
**Precipitate M3 subfraction or BrainUp-10^®^ elicited RHN cell morphological changes at a wide range of concentrations**. Concentrations from 0.5 μg/mL to 5.0 μg/mL of both the Precipitate M3 subfraction and BrainUp10^®^ were tested in RHN cells (*n* = 3). Concentrations of 0.5 and 5.0 μg/mL of Precipitate M3 subfraction resulted in an increased neurite length in RHN; meanwhile, the same effect was seen with 0.5 and 1.0 μg/mL of BrainUp-10^®^. Values represent the standard error. ANOVA test, factor followed by post hoc Tukey test. * = *p*-value < 0.05, *** = *p*-value < 0.001.

Due to the potent effects of Precipitate M3 subfraction on N2a cell models, we conducted further analyses with this subfraction and BrainUp10^®^ on RHN cells, evaluating morphological changes in the number and length of neuronal processes (Figure 8). Both the Precipitate M3 subfraction and BrainUp10^®^ increased neurite length at a concentration of 0.5 μg/mL. However, no significant changes in the number of processes were observed (Figure 9).

### 2.5. AnSh Fractions Inhibit Human Tau Aggregation

**Figure 10:**
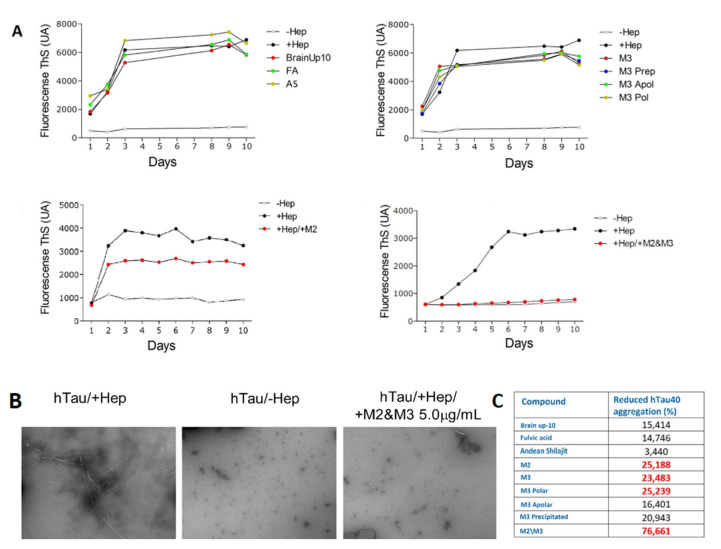
**Representative effect of AnSh fractions on the aggregation in vitro of recombinant hTau 40 protein**. Concentrations of 5 μg/mL of each AnSh M3 subfraction, M2, and M3 fractions (individually or in combination), fulvic acid (FA), Andean Shilajit (AnSh), and BrainUp-10^®^ were tested (*n* = 1). (**A**) Representative tracing curves of aggregation of hTau 40 during 10 days of registration, with different treatments performed through Thioflavin S (ThS) fluorescence assay. (**B**) Representative images acquired by electron microscopy (magnification 25,000× *g*) of hTau 40 aggregation in the presence (+Hep, maximum) and absence (−Hep, minimum) of Heparin. (**C**) Inhibition percentage of hTau 40 aggregation obtained with different treatments. Highlighted are the values with the highest inhibition percentage.

All AnSh fractions were evaluated for their anti-tau aggregation effect, using human recombinant tau protein (htau40) and ThS as protein aggregation markers. Nearly every AnSh fraction inhibited tau aggregation. Due to their previously observed strong neuritogenic effects, we aimed to test M2 and M3 alone and in combination to test for synergistic effects. Moreover, our most potent fraction was the mixture of M2/M3 at 0.5 μg/mL, which almost completely inhibited tau aggregation (Figure 10). BrainUp-10^®^ (15.4%), fulvic acid (14.7), and Andean Shilajit (3.4%) showed lower anti-aggregation potency. Even the Polar (16.4%) and Apolar (20.9%) M3 subfractions showed higher aggregation effects than the control group (heparin).

### 2.6. Characterization of Two Main Candidates by UPLC/MS

The AnSh fractions used in biological studies were obtained with different yields (0.036–6.0%). The largest yields were rich in fulvic acid (2.1%), to the M3 fraction (0.6%), and within this, to the Precipitate M3 subfraction (6.0%).

**Figure 11:**
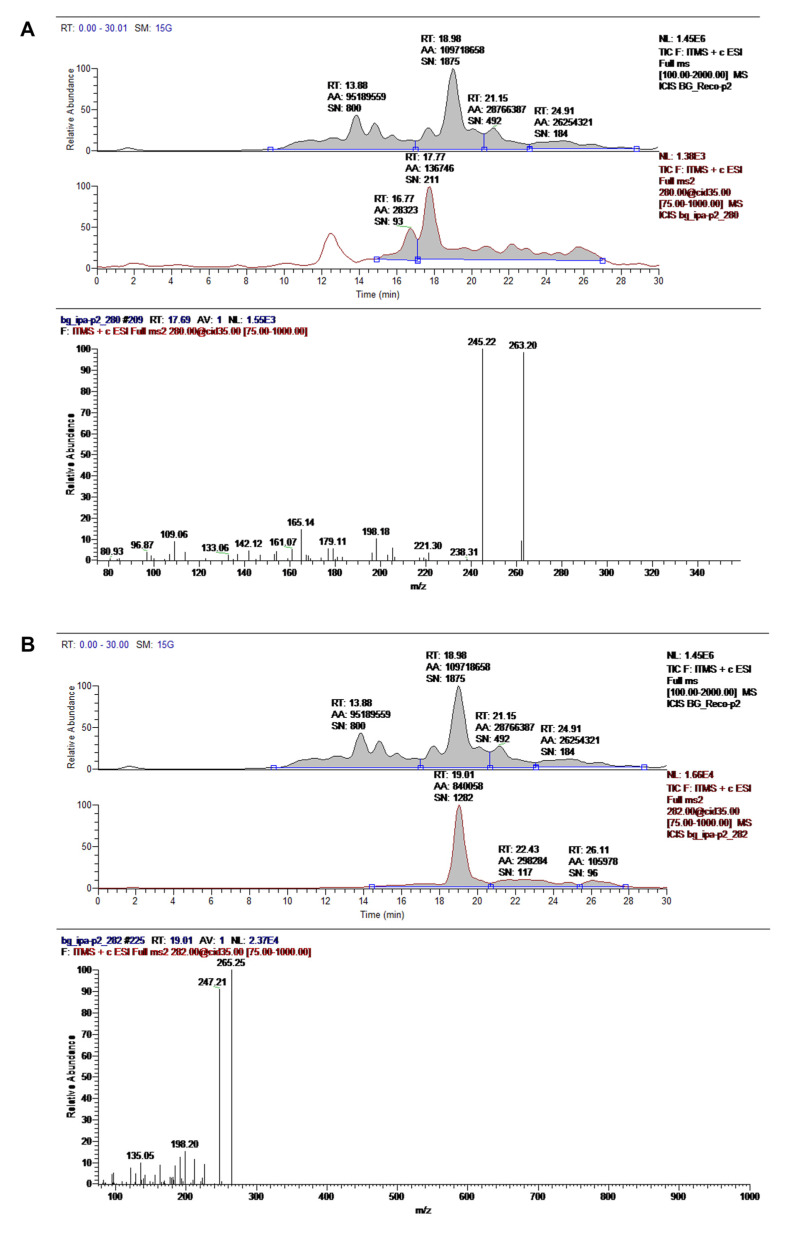
**Main compounds characterized by UPLC/MS in the M3 fraction precipitate.** The chemical identities of the dipeptide (**A**) and ASM3 (**B**) were assigned based on the chromatographic profiles of the UV and MS spectra. Records were evaluated at 35.0 eV.

Two novel therapeutic candidate molecules were identified, a dipeptide (Figure 11A) and a compound structurally similar to an oleamide that we denominated as ASM3 (Figure 11B) from the most promising M3 fraction precipitate, according to the morphological changes observed in RHN and N2a cells. These candidate compounds characterized by UPLC/MS were evaluated at 35.0 eV and are currently being studied for further application by our group.

## 3. Discussion

Currently, only a few drugs have been approved for clinical use against memory loss and cognitive disabilities; however, chronic treatment with these drugs is associated with low efficacy, and fails to reverse the course of AD and other dementias [33,34]. The high percentages of failures in clinical trials against AD have motivated several research groups to change their therapeutic strategy to focus on preventing AD and other neurodegenerative diseases. The new approaches for preventing AD are based on changes in lifestyle, diet, and the use of nutritional supplements, functional foods, and nutraceuticals [12,13,14]. In this context, our laboratory has thoroughly researched the Andean Shilajit and clinically validated the BrainUp-10^®^ formula, which is based on Andean Shilajit and B complex vitamins [12,22,23,24]. 

In this study, we evaluated the activity of Andean Shilajit and, for the first time, we reported the effects of its novel fractions on neuritogenesis and aggregation of human tau protein, in consideration of the fact that the damage to neurons, synaptic transmission, and, consequently, to memory processes induced by tau protein aggregation is central to developing AD. We analyzed the neuroprotective effect on the formation and elongation of neurites using the model of neuroblastoma N2a cells [27,29,35]. Fractionating the Andean Shilajit allowed us to identify that M2 and M3 fractions induced growth and elongation of neurites, with the most promising effect coming from the Precipitate M3 subfraction (60% greater than M2). Hence, Andean Shilajit and the BrainUp-10^®^ formula containing enriched fractions increase neurites growth and other cellular prolongations. The most potent fraction was Precipitate M3, which promoted the enlargement of the neuronal processes in both N2a cells and the primary culture of RHN. This effect was not evident in the unfractionated Andean Shilajit or the BrainUp-10^®^ formula, suggesting that one or more molecules may be diluted within AnSh or display antagonistic effects when interacting with other metabolites. Taken together, our data confirm the increased effect of these novel fractions compared to AnSh or BrainUp-10^®^.

The main constituent of Andean Shilajit and BrainUp-10^®^ is fulvic acid, a water-soluble compound endowed with potent antioxidant, anti-inflammatory, immunomodulatory, and neuroprotective effects through the inhibition of tau protein aggregation [36,37,38]. According to our results, the fulvic acid present in both Andean Shilajit and the BrainUp-10^®^ formula could be one of the bioactive compounds inducing neuritogenic and axogenic activity [22]. Previous studies have already suggested that BrainUp-10^®^ promotes neuritogenesis on hippocampal cells [12,23]; these data and our findings demonstrate its efficacy at controlling AD pathological processes [22,23]. Moreover, our results suggest that Andean Shilajit might contain other compounds with neuroprotective effects, and these compounds seem to act synergistically with other molecules, such as B-complex vitamins, enhancing the BrainUp-10^®^ formula [12,22]. 

Protein misfolding and protein aggregation are known to greatly impact the pathophysiology, diagnosis, and progression of AD [39,40]. There are different theories attempting to explain the origin of AD, the neurodegenerative events that lead to memory loss, and the formation of intracellular neurofibrillary tangles induced by hyperphosphorylated tau protein [40,41]. Tau is essential to establishing synaptic plasticity in memory and learning processes [42,43,44]. Some groups have proposed tau aggregation as a “druggable” target for AD, since several studies have confirmed a strong correlation between tau aggregates and cognitive decline [39,41]. The pathological forms of tau trigger an immunomodulatory positive feedback loop, where microglial cells are activated, releasing proinflammatory molecules that, as a consequence, promote tau aggregation [11,45,46].

Due to its relevance, we evaluated the effect of Andean Shilajit and its fractions on disentangling pathological in vitro tau aggregates. We used the ThS fluorescence method to indirectly quantify the formation of tau fibril aggregates [47,48], and we also corroborated our findings through electronic microscopy. Unfractionated Andean Shilajit produced a discrete inhibition of tau filament structures, as well as the disassembly of already-formed tau polymers. Our results demonstrated that fulvic acid prevents tau aggregation [36]. Likewise, previous studies have also shown the neuroprotective role of BrainUp-10^®^ at preventing tau aggregation in in vitro systems [23]. However, the strongest anti-aggregative effect was observed using the novel M2 and M3 fractions of the Andean Shilajit; in particular, the M2/M3 combination inhibited tau aggregation by 76.6%. These effects could be attributed to several compounds in a single fraction, or the result of combined actions with humic substances (humins) present in fairly high proportions in the Andean Shilajit [36]. Humins (fulvic and humic acids) are known to display neuroprotective properties, at molecular and clinical levels, where humins have been suggested to ameliorate the incidence of neurodegenerative effects. The synergistic effects of these acids with other molecules on each fraction could explain the outcomes observed in our experiments. 

The molecular characterization of the Andean Shilajit and its fractions revealed two main structures: the ASM3, which is chemically similar to an oleamide compound, and a dipeptide linked by an amide bond. Both dipeptides and oleamides have previously been tested in neuronal cultures and animal models, where they demonstrated neuroprotective effects and modulation of several nervous processes. Oleamide (cis-9,10-octadecenoamide) is a fatty acid amide with centric action that belongs to the family of endogenous lipid signaling molecules that includes endocannabinoids [49,50]. It was first found in the cerebrospinal fluid of sleep-deprived animals, referred to as acting as an endogenous sleep-inducing substance [51]. In addition, systemic administration of exogenous oleamide has been shown to induce various effects on the central nervous system (CNS), including memory facilitation [52,53,54], improving neuronal synapse function [54], and reducing epileptic activity [55]. Recently, oleamide has been shown to reduce the accumulation of Aβ by promoting microglial phagocytosis processes and suppressing inflammation post deposition [56]. In this context, we suggest that the presence of oleamide in subfractions of the Andean Shilajit provides strong neuroprotective and memory-enhancing properties, as well as presumably in BrainUp-10^®^ [54]. Nam, H. Y and collaborators demonstrated that oleamide blocks the cleavage of CRMP-2 (collapsin response mediator protein 2) through the inhibition of calpain activity. CRMP-2 plays a central role in neuronal differentiation, control of neuronal polarity, and axonal growth from neurites. Additionally, it has been related to NFT formation and degenerated neurites in AD and other dementias, so it is currently a promising target for the study and treatment of AD [57,58].

Moreover, we identified a dipeptide in the M3 fraction which could be a key component in the molecular mechanisms that mediate the growth and elongation of neurites in N2a cells and the aggregation or metabolism of tau protein aggregates. Large projects have emerged in recent years aimed at developing dipeptide-based pharmaceuticals. The advantages of dipeptides over longer peptides are that they are orally active due to their greater stability and their ability to penetrate biological barriers due to the presence of specific ATP-dependent transporters in enterocytes and the blood–brain barrier [59,60]. For stereo-chemical reasons, small peptide side-chains can be better immersed in the receptor cavity to establish a peptide–receptor interaction, which is a key element in the theoretical basis for the design of products based on di-, tri-, and tetrapeptides [59]. Such is the case in the Piracetam analog Noopept, which is under study for its potential as a memory enhancer and neuroprotector [59,61]. The dipeptide pGlu-Phe has been reported to inhibit the release of nitric oxide, tumor necrosis factor α, and interleukin 6, which are inflammatory mediators [62]. Meanwhile, Asn has been associated with the release of GLP-1 (glucagon-like peptide-1), a mechanism that has been proposed as a potential reducer of the accumulation of Aβ and its cytotoxic effects in an AD model [63], as well as a controller of insulin resistance, neuroinflammation, neurogenesis, neurite growth in the brain, and memory protection [64]. 

The cellular viability studies in the N2a cell model support the safety of our novel AnSh fractions at different concentrations. All of our findings suggest that these novel bioactive compounds identified in Andean Shilajit fractions can induce a positive modulation of neuronal function. Moreover, these compounds could act in vivo on aggregates of tau protein, turning off their positive feedback loop mechanisms, which will in turn stop the inflammatory cascade by the microglial cells, reducing the number of affected synapses and subsequent neuronal death. Finally, our article provides new evidence on the chemistry of AnSh by identifying, for the first time, the structure of bioactive molecules in this material. Another innovative aspect of this article is the mode of action of novel purified bioactive fractions of AnSh. We describe the protective effects of specific novel fractions and compare them against AnSh and BrainUp-10^®^. These pharmacological effects have not previously been reported in the literature, and present promising novel bioactive compounds for further research toward new pharmacological agents or nutraceuticals against Alzheimer’s disease, and potentially other neurological diseases.

## 4. Materials and Methods

### 4.1. Andean Shilajit

The different compounds, standards, and bioactive formulations used in these studies were obtained and provided in the framework of a collaboration between the different entities of the authors, mainly the University of Chile, the University of Santiago de Chile, and the International Center for Biomedicine.

### 4.2. Liquid Chromatography–Electrospray Ionization Mass Spectrometry (LC-ESI–MS)

The assay was performed using an LTQ XL linear ion trap (ThermoScientific, MA, USA) interfaced with a Thermo Scientific UHPLC system equipped with a quaternary pump (UltiMate 3000 High-Speed LC System). The ESI source was set in positive and negative ion detection modes. Full-scan MS data were collected for a mass range of 100–2000 *m*/*z*. The sample separation by ESI (+) was conducted on Intertsil^®^ ODS-4 HP column (2.1 ID × 100 mm, 3 μm, 100Å). Chromatographic analyses were carried out using the gradient method with formic acid (0.1% in MiliQ water) as eluent A and formic acid (0.1% in ACN J. T. Baker) as eluent B, with time (t) as follows: t = 0–1 min hold 98% A to 5% B, flow 0.2mL/min; t = 1–15 min, ramp linearly to 20% A to 80% B; t = 15–22 min hold 20% A to 80% B; t = 22–25 min ramp linearly to 95% A to 5% B; t = 25–30 min hold 95% A to 5% B. Sample separation by ESI (-) sample separation was conducted on a Kinetex^®^ column (100 × 3mm, 1.7 μm, 100Å). Chromatographic analyses were carried out using the gradient method with ammonium acetate (20mM in MiliQ water) as eluent A and ACN J. T. Baker as eluent B, with time (t) as follows: t = 0–1 min hold 98% A to 5% B, flow 0.2mL/min; t = 1–15 min, ramp linearly to 20% A to 80% B; t = 15–22 min hold 20% A to 80% B; t = 22–25 min ramp linearly to 95% A to 5% B; t = 25–30 min hold 95%.

### 4.3. Mouse Neuroblastoma Cells

Mouse neuroblastoma cells (N2a) were cultured in 10.0 cm plates. Each plate contained a final volume of 10.0 ml of DMEM Full medium (Gibco, New York, NY, USA) containing 10% FBS (Gibco, New York, NY, USA) and 1% penicillin/streptomycin (Sigma Aldrich, West Springfield, MA, USA). Cells were maintained for 48 h and incubated at 37 °C in a humidified 5% CO_2_ incubator, obtaining 80–90% confluence.

### 4.4. Primary Cultures of Rat Hippocampal Neurons (RHN)

Hippocampal neurons were dissociated and maintained as described previously [65]. Briefly, neurons were taken from 18-days-pregnant Sprague–Dawley rats and maintained for 14 days in vitro (DIV) on 12-well culture plates (500,000 cells/well) coated with poly L-lysine (Sigma, West Springfield, MA, USA) and supplemented with neurobasal/B27 media (Gibco, NY, USA). The culture was placed on a shelf in a 37 °C humidified CO_2_ incubator and the medium was changed every 2 days. Interventional studies involving animals received ethical approval (18150-FCS-UCH, date 29 May 2018) from the University of Chile. 

### 4.5. Viability Assay

N2a cells were seeded in 96-well plates with a confluence of 7000 cells/well. On the next day, cells were treated with variable concentrations of the bioactive compounds (0.1–100.0 µg/mL) for 24 h, at 37 °C and 5% CO_2_. Control groups were: (1) Cells without the treatment; and (2) a blank control, in which dish wells contained the medium but no cells. Cell viability was measured by the levels of blue formazan products formed from the colorless 3-(4,5-dimethyl-2-thiazolyl)-2,5-diphenyl-2H-tetrazolium bromide (MTT) by mitochondrial dehydrogenases, which are only active in viable cells. The medium was replaced by fresh DMEM medium without phenol red, and 10 µL of MTT [12 µM] was added and incubated for 4 h at 37 °C and 5% CO2. Then, 100 µL of 5% SDS-HCl solution was added and incubated under the same conditions for 4 h, and the absorbance was recorded at 550 nm using a Sunrise plate reader (Tecan, ZRH, Switzerland).

### 4.6. Immunofluorescence

N2a cells were plated on glass coverslips (12mm diameter; 10,000 cells/well). After being exposed to the experimental conditions, the cells were washed with PBS and fixed with paraformaldehyde–sucrose (4%) for 30 min at 37 °C. Cells were blocked with 5% BSA in PBS and then incubated with mouse alpha-tubulin 1:1500 (Invitrogen, MA, USA), TO-PRO 1:500 (Life Technologies, CA, USA) to label nuclei, Alexa Fluor-488 phalloidin at 1:300 (Invitrogen) for staining actin filaments and goat anti-mouse AlexaFluor-555-conjugated 1:400 (Invitrogen). Slides were mounted in FluorSave fluorescence mounting media (Calbiochem, CA, USA), and images were collected with a Carl-Zeiss Laser Scanning Confocal Microscope. The photographs obtained were representative of the different samples. The length and number of neurites were evaluated for post-incubation and immunofluorescence assays using the LSM Image Browser software (v. 4.2), and GraphPad Prism (v. 5.03) was used to generate graphs and perform statistical evaluation.

### 4.7. Tau Protein Purification

Tau protein purification was carried out as previously described [65]. The sequence of htau 40 (longest isoform) was generously donated by Dr. Eckard Mandelkow (Hamburg, Germany). This fragment was cloned into a pET-28ª vector (Novagen, PSA) to produce a His-tagged protein, and htau 40 was expressed in *Escherichia coli* strain BL21(DE3). LB medium containing kanamycin was inoculated with a stationary overnight culture. The culture was grown at 37 °C to OD_600_ of 0.5–0.6 and protein expression was induced by the addition of 1mM IPTG for 4 h. The cells were pelleted and sonicated. Recombinant tau was purified via successive Ni-Sepharose chromatography (equilibrated in 20 mM NaH_2_PO_4_, 500 mM NaCl, and 20 mM imidazole, pH 7.4, elution with buffer 200 mM imidazole) and size exclusion chromatography coupled to HPLC in a Proteema 100 column (PSS, Germany) with buffer 50 mM NaH_2_PO_4_, 300 mM NaCl, pH 6.5. The purity of the protein was verified on a Coomassie Brilliant Blue-stained SDS-polyacrylamide gel (12%), detected with the antibody anti-htau4, and analyzed on the basis of densitometry using ImageJ software (version 1.52) [66]. The protein was concentrated and stored at –20 °C until use. The concentration of purified tau was determined using the extinction coefficient at 280 nm (7700 M ^− 1^ cm ^− 1^).

### 4.8. Tau Aggregation Assays

The aggregation of htau40 for the experiments was achieved by incubating htau40 at a concentration of 50 µM in a final volume of 1 ml at 37 °C in PBS buffer at pH 7.4 with the anionic cofactor heparin (molar ratio of tau to heparin = 4:1) for an incubation time of 8 days, under continuous shaking. In the case of the control, the protein was incubated at the same concentration without the aggregating agent heparin.

### 4.9. ThS Fluorescence Assay

The htau40 aggregation was monitored on the basis of the fluorescence of Thioflavin S (ThS), as described by Friedhoff [48] and Barghorn [67]. For Tau aggregation, to the htau40 and heparin solution, ThS (6.25 µM) was added and incubated for 1 h at 37 °C in the dark and stirring to allow binding of the fluorescent probe with the structures formed on the tau beta-sheet. Fluorescence was measured on a Biotek Synergy 2 computer with an excitation of 440 nm and emission at 508 nm (excitation filter 440/30 and emission filter 508/20), minus the basal fluorescence of a blank incubated under the same conditions. In vitro experiments were performed using purified recombinant hTAU40 protein aggregation in a workbench station fluorimeter for 10 days with a formulation of an Andean Shilajit fraction. At the end of the aggregation process, the molecular aggregates were visualized by electron microscopy using a JOEL EM1200 microscope.

### 4.10. Statistical Analysis

The analysis was performed using GraphPad Software, version 5.00 (GraphPad Software Inc., La Jolla, CA, United States). The results are expressed as mean ± SEM. Data were analyzed by one-way analysis of variance (ANOVA) followed by Tukey’s post hoc test for multiple comparisons. A value of *p* < 0.05 was considered to be statistically significant. Additional details are provided in the figure legends, where appropriate.

## 5. Conclusions

Our results indicate that the Andean Shilajit and its fractions can modulate neuronal function and promote neuritogenic effects (in terms of both length and number of processes). At the same time, they do not impair cell viability. Additionally, they can promote an anti-aggregative effect and the disassembly of tangles and oligomers of tau protein, suggesting their potential for the treatment of neurodegenerative diseases such as AD. The molecular characterization of fractions unveiled two major candidates for therapeutic approaches, which will be subjected to further studies using new experimental approaches to understand the molecular roles involved in these effects. Finally, this will promote the development of further research on the efficacy and safety of natural compounds in the onset and/or progression of neurodegenerative and neurodevelopmental disorders.

## Figures and Tables

**Table 1:** Effect on the length and number of neurites in N2a cells mediated by different fractions and subfractions of Andean Shilajit.

Compound/Fraction	Neurite Length Variation (%)			Neurite Number Variation (%)		
	0.5 µg/mL	1.0 µg/mL	5.0 µg/mL	0.5 µg/mL	1.0 µg/mL	5.0 µg/mL
**M1**	12.986	13.097	27.252 **	34.493	71.125 *	13.849
**M2**	38.536 *	11.781	24.360	42.047	33.258	62.826 *
**M3**	16.240	20.424	35.233 *	−17.777	−30.791	−16.643
**Polar M3**	21.470	23.782	35.728 *	14.545	−0.657	0.022
**Apolar M3**	31.241	26.479	34.187	3.204	−14.834	−28.331
**Precipitate M3**	63.198 **	34.930 *	42.609 *	47.434	70.224 *	68.069 *
**M4**	−19.942	−7.420	1.159	11.487	−13.040	−8.933
**Andean Shilajit**	−9.909	−9.744	−8.671	31.557	2.178	28.375
**Fulvic Acid**	22.956	16.295	26.865	59.909 *	10.684	68.069 *

Bold values exhibit significant differences (* = *p*-value < 0.05, ** = *p*-value < 0.01), with control as reference.

## Data Availability

Data is contained within the article.
